# Retraction Note: H3K27ac-induced lncRNA PAXIP1-AS1 promotes cell proliferation, migration, EMT and apoptosis in ovarian cancer by targeting miR-6744-5p/PCBP2 axis

**DOI:** 10.1186/s13048-023-01327-7

**Published:** 2023-12-19

**Authors:** Yimin Ma, Wei Zheng

**Affiliations:** 1 Department of Gynecology, Ningbo Medical Center Lihuili Hospital, Ningbo, 315040 Zhejiang China; 2 Department of Gynecology, Xi’an Military Industry Hospital, Xi’an, 710065 Shaanxi China

**Retraction Note: Journal of Ovarian Research (2021) 14:76**

10.1186/s13048-021-00822-z

The authors have retracted this article. After publication, concerns were raised regarding image overlap between Fig. 5 of this article and Fig. 1 in [1], which the authors addressed by a Correction [2]. However, in follow-up experiments, the authors were unable to replicate the results of this study, which undermined the conclusions of this article.

All authors agree to this retraction.
